# Evidence-based information about intermittent fasting in diabetes patients: useful or harmful?

**DOI:** 10.55730/1300-0144.5386

**Published:** 2022-03-26

**Authors:** Mustafa ALTAY

**Affiliations:** Department of Endocrinology and Metabolism, University of Health Sciences Turkey, Retired Lecturer, Ankara, Turkey

**Keywords:** Calorie restriction, diet, glucose reduction, intermittent fasting, type 2 diabetes mellitus, weight loss

## Abstract

**Background/aim:**

One of the most important components of treatment for diabetic patients is diet and healthy nutrition therapy. Calorie restriction is effective and without cost increases its appeal for both patients and physicians. Unfortunately, continuous calorie restriction is a difficult method. For this reason, alternative calorie restriction methods, such as intermittent fasting (IF), have been investigated by some researchers.

**Materials and methods:**

IF refers to a wide range of diet programmes covering periods of eating and fasting, which vary according to the different regimens. In this article, first, some general information will enable us to understand the concept of IF, and then scientific evidence with respect to IF applications in diabetes will be discussed in detail. Thereafter our clinical experience will be summarised, finally, the author will try to answer the question “are the IF applications beneficial or harmful for diabetic patients?”

**Results:**

Considering animal studies, epidemiological studies, pilot studies, clinical experiences and a small number of randomized controlled trials conducted so far, it seems possible to say that the beneficial effects of IF for diabetes patients are greater than potential harms. However, there are not yet enough studies with a high level of evidence to recommend IF as a routine part of the treatment in patients with diabetes.

**Conclusion:**

It is necessary to show which IF regimen is safe and effective, how often and for how long, for diabetic patients. This seems possible with well-designed randomized controlled trials focusing on long-term clinical outcomes and eliminating confounding factors. This will make the answer clearer.

## 1. Introduction

The causal relationship between type 2 diabetes and obesity is well known. In fact, this association has been called a “diabesity” epidemic in recent years. The effect of weight loss on blood glucose regulation and on many metabolic events has been proven. There are also many studies demonstrating its effects on the long-term course of diabetes as well as complications [1,2]. Therefore, one of the most important components of treatment for diabetic patients is diet and healthy nutrition therapy. In recent years, the search for alternative applications to limit calorie intake has come to the fore when determining weight loss strategies for patients with diabetes [1]. Because continuous energy restriction application is a difficult method, intermittent fasting (IF) has emerged as a method which has been considered, researched and applied by some authors. The most significant contribution to the subject has been in the area of Ramadan fasting. The fact that calorie restriction (CR) is effective and without cost increases its appeal for both patients and physicians and/or researchers.

In this article, first, some general information will enable us to understand the concept of IF, and then scientific evidence with respect to IF applications in diabetes will be discussed in detail. Ramadan fasting is accepted as a type of IF, but since it involves some differences in terms of diurnal rhythm, changes in sleep patterns, eating times and content, we will only discuss some general information about Ramadan fasting, and Ramadan fasting-diabetes studies will not be detailed. We published our book with detailed information on Ramadan fasting and diabetes in Turkey [4].

## 2. Intermittent fasting

Since almost all religions include the concept of fasting, IF is a lifestyle that has been practiced since human existence began. When we look at the modern medical literature, we see that animal studies on IF started in the first half of the twentieth century [4, 5], and the first human studies with IF were carried out at the beginning of the second half of the century [6]. After the 1990s, IF became a popular research topic.

Human beings have biological rhythms, many of these physiological and behavioural rhythms being circadian rhythms, following an approximately 24-h cycle. From this point of view, nutrition also has timing, and this will affect the eating/fasting periods. Ultimately, the metabolic rhythm will be affected, and insulin sensitivity, glycaemic control and weight balance will change. It has been shown in animal studies that metabolic pathways are negatively affected by irregular and disrupted meal programmes [7]. It is also known that metabolic disorders such as obesity, insulin resistance and diabetes develop with disruptions in eating/fasting cycles in gene disorders related to the mechanisms of the circadian clock.

IF refers to a wide range of diet programmes covering periods of eating and fasting, which vary according to the different regimens. The fasting period is usually over 12 h. During these periods, the person may be free to consume calorie-free liquids such as water and tea, and while calories are restricted in some regimens during eating periods, in others, the person takes food as desired [5]. The expected effects of IF on the body and the metabolism first occur during periods of fasting and continue as the process progresses.

In the feeding model called intermittent energy restriction, over a period of approximately 24 h, the person receives 25%–40% of the energy to be taken daily, and then there are periods of normal food intake; the timing of these periods can differ. These are followed by energy-constrained time periods, with the two periods alternating with each other [5]. Although this method is used in the literature in the same sense as IF, there is no complete fasting period in these regimens.

Table shows the intermittent nutrition models used in animal experiments and human studies conducted so far. Among these, the 16/8 diet and the 5:2 diet are most commonly used in clinical studies and are the most popular diet regimens applied in daily life.

Animal studies have found that IF has positive effects on atherosclerosis [8], cognitive functions [9,10], the immune system [11] and the metabolism [8,9] and that it extends the lifespan [12,13]. There are also clinical trials, weight loss of up to 8%–10% and significant improvements in LDL-C, TG and CRP compared to the control group are reported with IF. The opinion is that the cellular-molecular effects which provide these metabolic changes occur through three main pathways. Path 1: AMP-activated protein kinase (AMPK) is activated as a result of the increase of AMP with the decrease of intracellular ATP. Path 2: Decreased circulating glucose and amino acids with fasting inhibit rapamycin (mTOR), the intracellular energy sensor [14]. Path 3: Due to the need for energy, glycolysis starts in the liver, with a fatty acid release from adipose tissue [15]. Health is positively affected by increasing autophagy and decreasing oxidative stress. As clinical reflections of these processes, weight loss, a decrease in insulin and blood glucose and a decrease in blood pressure and serum lipid levels occur.

Mild side effects have been reported, especially in the first days of IF application. These include weakness, headache, muscle weakness, signs of dehydration, hypoglycaemia, gout and peptic ulcer activation [1,16]. Situations such as anxiety and psychological stress were also reported in some studies [17]. Although these complaints are generally well tolerated, it is an appropriate approach to avoid IF for patients, for example, with an active peptic ulcer or upper GIS bleeding as well as for type 1 diabetes patients who can easily enter hypoglycaemia, for the elderly, for those who cannot tolerate dehydration and for those with malnutrition. Side effects such as postural hypotension, gout and cardiac arrhythmia are mostly associated with prolonged fasting-starvation and have not been reported frequently in IF studies.

Clinical studies conducted with IF in people with chronic diseases which cause comorbidity, such as coronary artery disease, chronic kidney disease and diabetes are almost absent. It should be kept in mind that the harmful effects which may occur in such patients may be more serious. More serious complications may also occur with not eating adequately or with lacking balance during periods of not fasting while applying IF, for example, drinking less water, reducing protein and not getting enough vitamins and minerals. Conditions such as stroke due to severe dehydration or muscle wasting due to excessive protein reduction can occur, especially in long-term and uncontrolled fasting regimens [16].

## 3. Intermittent fasting and diabetes-related animal studies

The animal studies concerning IF and diabetes are mostly focused on the effects of IF on pancreas beta cells, by using either in vivo or in vitro techniques. Murine modelling has generally been used in animal studies investigating the effects of intermittent fasting on diabetes. For diabetes modelling, some studies used a hypercaloric high-fat diet, while some used leptin receptor knock-out mice [17]. Fast-mimicking diet (very low calorie diet), alternate day fasting and time restricted feeding are generally applied as an IF protocol [17]. The most important common finding detected in animal models is the improvement of beta cell function and insulin resistance without weight loss. It has been shown that the lysosome-autophagy and neurogenin 3 (Ngn3) pathway play a role in these effects [17]. By the decrease of blood glucose after fasting, autophagy-mediated suppression of Notch 1 signalling occurs. Thereafter protein Ngn3 (progenitor pancreatic cells) activation happens in pancreatic islet cells, and leads to beta cell proliferation and neogenesis [18]. Thus, beta cell mass and insulin secretion increase. In damaged beta cells, this activation does not occur, and the process results in autophagy [19].

In many animal studies using both calorie restriction and different regimens of IF, positive effects such as increased insulin sensitivity, improvement in lipid parameters, weight loss, shrinkage of adipose tissue and regression of systemic inflammation have been shown [17,20]. Furthermore, a mouse study had shown that the expected positive effects on beta cells were seen additionally, and the microbiota was also found to be improved with IF [21].

No adverse event or complication has been seen in diabetic mice with IF in animal studies published up to date.

## 4. Clinical studies on intermittent fasting and diabetes

Generally, the studies on IF and diabetes up to now have investigated about effects on peripheral insulin resistance and glucose homeostasis and how these effects manifested clinically. Thus, in vitro studies have not been performed yet.

The metabolic effects caused by IF administration in experimental animal models have been investigated. It has been reported that a “*metabolic switch*” occurs with IF, and the activation of many pathways occurs with it [15]. It is thought that these mechanisms of action are also valid in humans and that beneficial metabolic effects occur in this way. Due to low blood glucose levels during fasting periods, less insulin secretion occurs compared to other times [22]. In addition, insulin peaks and cortisol release that occurs in diurnal nutrition with IF become more suitable for the physiological pattern. These effects contribute significantly to glucose control [23,24]. It has also been reported that growth hormone increases with hunger. Although it decreases when the person eats again, it is claimed to contribute to the long-term health protective effect [25]. Increased serum ghrelin levels and decreased leptin concentrations have been reported in various IF studies [24,26]. It has also been reported that with IF, the oxygen carrying capacity increases with the increases in erythrocyte count and haemoglobin level, and as a result, metabolic functions improve and insulin resistance decreases [27]. It has also been reported that appetite is suppressed, and the feeling of satiety increases with IF [26].

Case reports have shown that weight, blood glucose and HbA1c control can be achieved and that IF applications were safe with regular IF administration for 7–14 months in individuals with diabetes and even comorbid diseases [28,29]. These reports noted that asymptomatic (mild) hypoglycaemia episodes had been seen, but the patients had been able to tolerate them and maintain IF without suffering. The patients also had revealed that they felt better and their life quality had improved. If we look at the IF and type 2 DM clinical studies in summary: Arnason et al. applied time-restricted feeding (18–20 h) for two weeks to 10 type 2 DM patients with obesity, treated with metformin. As a result, there was a decrease in blood glucose and the weight of the patients at the end of the period of IF administration, but no significant change was found in HOMA-IR and serum lipid levels [30]. In this short-term observational study, IF administration was reported to be safe for diabetes because any complication, including hypoglycaemia had not been observed. Kahleova et al. randomized 54 obese patients with good blood glucose control with oral antidiabetic agents into two groups with a similar calorie intake [31]. The first group received a diet consisting of three main meals and three snacks, and the second group received a B2 diet for 12 weeks. As a result, the researchers found significant weight loss and a significant decrease in hepatic adipose tissue, blood glucose, C-peptide, fasting insulin and glucagon levels only in the B2 group. This study did not report any adverse event, as well.

To give a few examples of intermittent energy restriction (IER) studies in diabetics: Carter et al. conducted a 12-week pilot study by randomizing 63 overweight and obese type 2 DM patients to a 5:2 diet and continuous energy restriction [26]. Eighty-one per cent of the patients completed the study. In the 12th week, a percentage of weight loss and a decrease in HbA1c occurred in both groups. The study reported that 6 patients using insulin and getting IF had hypoglycaemia and its frequency was not different from control group. Also, it was noted that hypoglycaemia had not repeated after insulin dose adjustment. Again, Carter et al. randomized 137 obese type 2 DM patients into a continuous energy restriction group and a 5:2 IER group for 52 weeks [32]. In the study, the calorie intake of both groups decreased by 30%. While the compliance of the patients with their diets was over 90% in the first 3 months, it later decreased to half of that. At the end of the study, a similar significant decrease in body weight, fasting glucose, HbA1c and LDL-cholesterol levels was found in both groups. These effects peaked in the third month and then continued to decrease. A significant reduction in drug doses was also detected in both groups. An approximate 50% reduction in insulin dose occurred in the IER group. Most dose adjustments in the study were made within the first three months. The more pronounced effect in these first three months was attributed to the high number of policlinic visits in that period. Hypo- and hyperglycaemic events were reported to be similar in both groups in the first two weeks of the study (mean 3.2 vs. 5.9 event, p = 0.28). In addition, two patients from IF group were reported to cease participation because of headache. The authors also published the 24-month follow-up results of the same study [33]. They found that weight loss continued at the end of the second year, but there was a 0.3% increase in HbA1c. These results were similar for both groups. The researchers commented that the increase in HbA1c is not related to diet but is related to the course of diabetes, as stated in previous large studies. They emphasized that the number of antidiabetic agents used by the patients was still less at the end of the second year compared to at the beginning of the study. No adverse event was reported. As a result, they stated that they showed that IER is at least as effective as continuous energy restriction in the long term.

Corley et al. applied the 5:2 diet with the consecutive day rule to 19 Type 2 DM patients for 12 weeks, and applied the 5:2 diet to 22 Type 2 DM patients on nonconsecutive days [34]. Significant weight loss, a lowering of fasting blood glucose and a decrease in HbA1c were observed in both groups. The reduction in blood pressure was not significant. Although the doses of antidiabetic drugs were reduced, an increased risk of hypoglycaemia was found, similar in both groups. However, the authors emphasized that IF is safe in diabetic patients, since severe hypoglycaemia was not observed. They attributed the risk of hypoglycaemia not to the IF type but to the person’s own characteristics. Authors emphasized that hypoglycaemia risk would be lower than expected by instruction and drug adjustment. Any other complication except hypoglycaemia was not reported.

Ash et al. reported that in obese diabetics, weight loss and glucose regulation was achieved with 12-week calorie restricted diets (IER, proportioned meals and self-selected meals), but this was independent of diet type [35]. In another study, three diets (five days of very low-calorie diet, one day of very low-calorie diet and standard behavioural diet therapy) were compared. Before the study, diabetes medications were discontinued for two weeks, and the patients were followed for 20 weeks. Weight loss and a decrease in fasting glucose levels were seen in the periodic very low-calorie diet groups. The decrease in insulin levels and lipid levels were similar in all three groups [36]. These studies also did not reported any adverse events.

In fact, in clinical practice, IF experience in patients with diabetes is the most common with Ramadan fasting, because most of the patients fast for the one month period of Ramadan, a religious duty in Islam around the world [4]. In a bibliometric analysis published in 2019, it was reported that 424 articles related to diabetes and Ramadan fasting in the 30 years between 1989–2018 were included in the literature [37]. For this reason, organizations such as the American Diabetes Association and the International Diabetes Federation and many countries have published guidelines on the management of patients with diabetes during Ramadan [38,39]. The point of view of the Ramadan fast, which Muslims observe for one month a year, should be evaluated slightly differently from other fasts, because it is known that the majority of Muslim diabetic patients around the world fast for at least 15 days in Ramadan. Here, patients fast because it is a religious obligation rather than because of the fast’s contribution to body health or diabetes management. Therefore, it is a correct approach to divide patients into very high-/high-/medium-/low-risk categories in terms of fasting and to make appropriate recommendations. Because uncontrolled and carelessly fasting can cause a risk for hypoglycaemia, hyperglycaemia, ketoacidosis and thrombo-embolism. After individual evaluations, it has been reported in many studies that patients with diabetes can safely lose weight, improve their lipids and provide glucose control with Ramadan fasting under conditions when the diabetic patient is appropriately enrolled before Ramadan, with their medication adjusted and with regular clinical blood glucose monitoring [4,38,39].

In the articles published so far, it has been reported that IF is generally safe for diabetic patients. It was emphasized that people using insulin and sulfonylurea should be alert because of the risk of hypoglycaemia [5,40]. Kahleova et al. [31] reported that the dose of insulin, sulpfonylurea and meglitinide should be reduced by 50% on fasting days in patients with type 2 diabetes, an action also suggested by Corley et al [34]. With this dose reduction, patients should still be closely monitored for hypoglycaemia. Some authors recommend changing the drugs according to the HbA1c result measured before IF. According to this recommendation, if HbA1c < 7, insulin, sulfonylurea and meglitinide should be discontinued, and if HbA1c is between 7 and 10, the dose should be reduced by 50%. If it is >10, the same doses should be continued [17,40]. Although the risk of hypoglycaemia is low in the use of metformin, thiazolidinedione, DPP-4 inhibitors, SGLT-2 inhibitors and GLP-1 analogues, one should be careful on fasting days [16]. During IF, it is recommended that glucose should be measured at home twice a day and that the diet should be stopped when severe hypoglycaemia occurs.

It should be mentioned that the IF concept and starvation are different. However, there are claims that if IF is performed in an uncontrolled way and for a long time, starvation, which is a catabolic process, may occur. In IF, since there is no frequent, long-term, excessive calorie restriction, as IF is applied in the therapeutic sense, symptoms seen in starvation, such as anaemia, diarrhoea, uncontrolled weight loss and delirium, are not observed.

The number of studies on IF in pregnant women with diabetes and on breastfeeding women are very few. These are also Ramadan fasting studies. Although the study results differ, generally, pregnant women (with or without diabetes) should not be recommended any IF regimen, including Ramadan fasting [4,16,39].

## 5. Our experience

Our practises are generally via as our approach on Ramadan fasting [4]. That is, we evaluate our patients before IF and see whether they are convenient to it. If the patient had not poor blood glucose regulation, experiences of hypoglycaemia or severe systemic complications, and had good orientation, we recommend a type of IF which he/she had adopted, under dietitian and clinician control. After the necessary instructions are completed and medications are adjusted, we firstly make them fast a few times and then contact to maintain routine IF procedure. Major part of our patient group carry out 2 days a week 16/8 diet and some of them everyday 16/8 regimen. We usually observe weight loss, improving blood glucose levels and lipid parameters in our patients. They do not have hypoglycaemia as soon as they go carefully. However, we have an important problem in our country that some of the patients do IF according to their own orientation and information they have got from internet and social media, without consulting with clinician or dietician. We observe complications like hypoglycaemia, malaise and headache at these people much more.

## 6. What is the answer?

Considering animal studies, epidemiological studies, pilot studies, clinical experiences and a small number of randomized controlled trials conducted so far, it seems possible to say that the beneficial effects of IF for diabetes patients are greater than potential harms. However, there are not yet enough studies with a high level of evidence to recommend IF as a routine part of the treatment in patients with diabetes. For patients who are willing to engage in IF, especially obese diabetics, if the clinical condition of the patient is also suitable, appropriate short-term IF regimens can be tried under the management of experienced physicians, nurses and dieticians. When making a decision, individual characteristics should be considered. Before starting IF, the patient should be adequately trained, and medications should be adjusted. The patient should also be followed closely with both clinical criteria and metabolic criteria such as BMI, blood pressure, glucose level and lipid levels. It is also necessary to be alert to complications, such as hypoglycaemia, fatigue, focus problems and hypotension, which may arise due to the possibility of uncontrolled fluid and energy restriction.

It is necessary to show which IF regimen is safe and effective, how often and for how long, for diabetic patients. This seems possible with well-designed randomized controlled trials focusing on long-term clinical outcomes and eliminating confounding factors. This will make the answer clearer.

## Figures and Tables

**Table:** Frequently applied diet regimens for calorie restriction.

Kind of diet regimen	Fasting duration (hours)	Frequency	Characteristics-considerations	Daily calorie restriction
**IF regimens**				
Time-restricted feeding (16/8)	14–18	Every day	The person determines the hunger time during the day	Voluntary
B2 regimen	16	Every day	2 main meals	Voluntary
Weekly one day fasting	24	Once a week (Every week)	Water only diet	Yes
Ramadan fasting	11–18	Voluntary (e.g., during Ramadan or any time during the year)	No any fluid or food (From dawn until sunset)	Voluntary
**IER regimens**				
Alternate day fasting	24	Every other day	Fasting day: 0%–25% of calorie Feasting day: Ad libitum	Yes
5:2 diet	24	Twice a week	Fasting day: consecutive or nonconsecutive Feasting days: ad libitum	Yes
Combination of IF and CR (IFCR)	24	Variable	e.g., six days calorie restriction and one day fasting	Yes
Intermittent VLCD	24	Variable	e.g., 1 day or 5 day VLCD	Yes

Abbreviations: IF: intermittent fasting, IER: intermittent energy restriction, CR: calorie restriction, VLCD: very low-calorie diet.
